# What are the important components of the clinical assessment of hand problems in older adults in primary care? Results of a Delphi study

**DOI:** 10.1186/1471-2474-11-178

**Published:** 2010-08-09

**Authors:** Helen Myers, Elaine Thomas, Krysia Dziedzic

**Affiliations:** 1 Arthritis Research UK Primary Care Centre, Primary Care Sciences, Keele University, Keele, Staffordshire, UK

## Abstract

**Background:**

To identify clinical questions and assessments regarded by health care practitioners as important when assessing undifferentiated hand pain or problems in adults aged 50 years and over presenting to primary care.

**Methods:**

A purposively selected panel of 26 UK-based Health Care Practitioners comprising occupational therapists, physiotherapists, rheumatologists and general practitioners, were invited to take part in a consensus study involving three postal rounds of a Delphi questionnaire with accompanying case scenarios. Participants were asked to generate questions and assessments (round 1), rate their importance (round 2), and vote on which items were most important (round 3).

**Results:**

Sixteen Health Care Practitioners agreed to participate with 11 completing all three rounds. The first round of the Delphi study generated 156 questions and 143 assessments. After three rounds agreement was reached on the importance of 25 questions and 19 assessments. Questions were weighted towards current symptoms, but also included the history of previous hand problems, self-reported hand function, co-morbidity and general health. Observation and palpation of features predominated in the choice of assessment, but specific tests, grip strength, evaluation of sensation and hand function were also included.

**Conclusions:**

A pool of clinical questions and assessments were generated by Health Care Practitioners, and those considered most important for assessing older adults presenting with undifferentiated hand pain and hand problems in primary care were identified. Further evaluation is required to establish the reliability and feasibility of using these questions and assessments in primary care. In particular, the relative contribution of these questions and assessments in evaluating the nature and severity of hand problems, assisting diagnosis, indicating appropriate management, and predicting future course requires further investigation.

## Background

Musculoskeletal hand pain is common in middle and old age, reported by between 12% and 30% of adults aged 50 years and over in the United Kingdom [1,2]. Most will self-manage without regular recourse to formal health services [3] but primary care is likely to play an important role in the initial and ongoing assessment for many, with one Dutch study estimating an annual incidence of new general practitioner consultations for hand complaints of between 4 and 12 per 1000 registered patients over the age of 50 years [4]. Previous studies suggest that in older people osteoarthritis is likely to be the most common diagnosis, with other specific conditions (for example, tenosynovitis, carpal tunnel syndrome) important but less common [2,5]. However, the clinical assessment must fulfil a range of other functions in addition to diagnosis, such as evaluating the nature and severity of the problem and its impact on the patient, predicting its likely future course, and selecting appropriate management. Given the multiple purposes of assessment, the relatively large spectrum of unspecified health problems presenting to general practice [6], and the often highly time-constrained setting [7], which questions and assessments are most useful remains unclear. There are many valid and reliable self-report measures for assessing the hand [8-10]. Hand assessment has also been included in examination schedules for the musculoskeletal system in general (Gait Arms Legs and Spine (GALS) schedule [11] and the Regional Examination of the Musculoskeletal System (REMS) for medical students [12]), and for the diagnosis, classification and assessment of specific hand disorders (the Southampton examination schedule [13] and the Sequential Occupational Dexterity Assessment (SODA) [14]). Despite the high prevalence of hand problems in older adults within primary care, little has been done to determine the best way to assess the hand, with the exception of Recht and colleagues [15]. In order to address the lack of literature in this area we conducted a consensus study involving UK health care practitioners from a variety of professional backgrounds with a role in the assessment and management of hand problems. As a range of health practitioners have expertise in hand assessment, and many of these work within secondary care settings, a variety of professional groups from a range of work settings were included in the consensus study. The specific aim of this study was to identify the clinical questions and assessments regarded by Health Care Practitioners as important when assessing undifferentiated hand pain or hand problems in adults aged 50 years and over and presenting to primary care. It is envisaged that the questions and assessments arising from this study and subsequent work could be used by any of the Health Care Practitioners involved in the care of the patient in primary care.

## Methods

### Participants

Participants from the UK were purposively sampled from four Health Care Practitioner (HCP) groups (occupational therapy, physiotherapy, general practice and rheumatology). Participants were identified by applying the following selection criteria: HCPs working in rheumatology and/or hand therapy, clinician, researcher or educator, and regularly using hand assessments (in practice, research or education). A convenience sample of occupational therapists and physiotherapists (Senior I grade or above) was identified from the membership lists of the College of Occupational Therapists Specialist Section-Rheumatology and the British Association of Hand Therapists. A convenience sample of consultant rheumatologists and general practitioners was identified from musculoskeletal publications within primary care. Twenty six HCPs were approached in writing and invited to participate. This number was chosen to allow for drop out at each stage, whilst balancing the requirement to have a minimum of ten participants by the end of the study to enhance reliability [16], with the need to avoid recruiting so many participants that large amounts of data were produced, making for long subsequent rounds and potentially increasing attrition.

### Methods

The Delphi technique was used to elicit the opinions of participating HCPs through successive rounds of a postal questionnaire [17]. Responses to each round of the Delphi were collated, analysed and the results returned to participants in the form of another questionnaire until an acceptable degree of consensus was achieved [16,18]. In the current study three rounds of a postal questionnaire with instructions for completion were sent to participants. For each round, participants who had not returned their questionnaire within two weeks were sent a reminder together with a repeat questionnaire. Non-responders to this reminder were excluded from further rounds of the study. Ethical approval for the study was granted by the North Staffordshire local research ethics committee (LREC No: 02/54).

**Figure 1:**
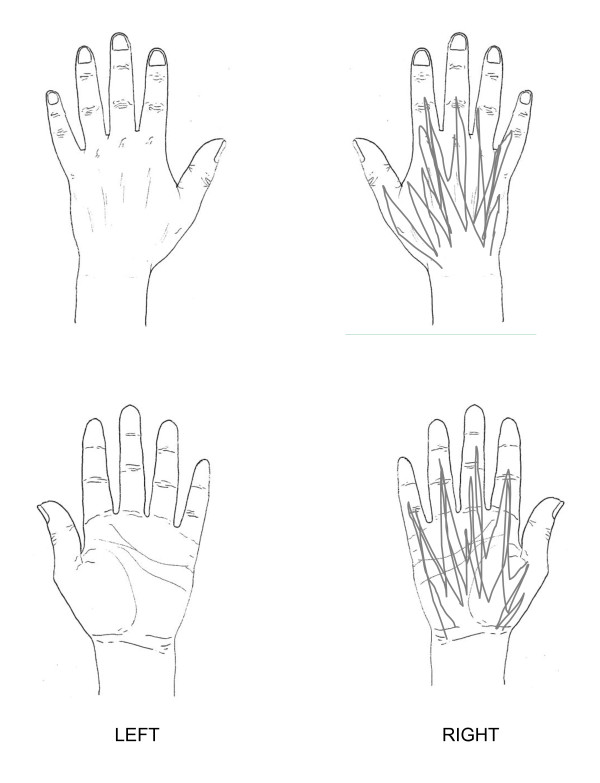
**Case scenario 1**. Mr Jones is a 51-year-old man who has recently presented to his GP with a 10-month history of right hand symptoms. Mr Jones is concerned that he is losing his independence and may have to give up his job as a joiner. On further questioning it emerges that he feels frustrated that he is becoming generally slower and clumsier. The drawing below indicates symptomatic areas shaded by Mr Jones.

**Figure 2:**
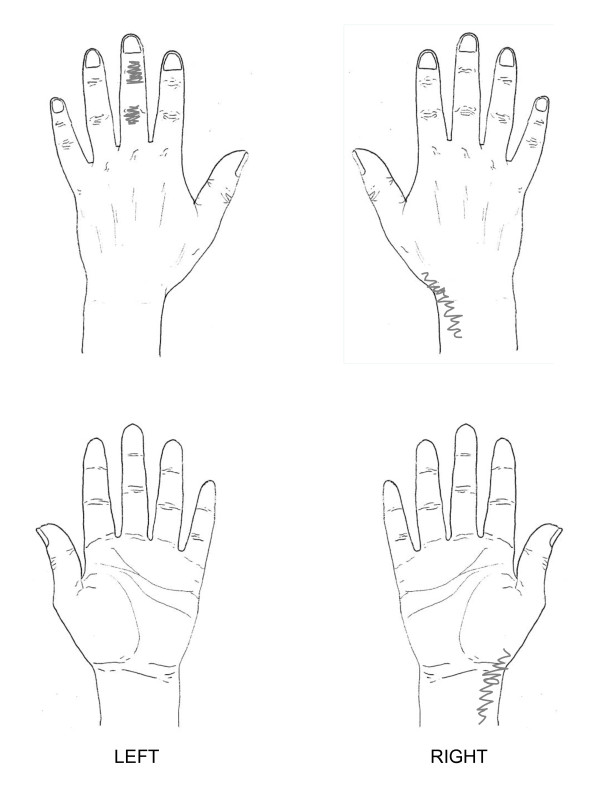
**Case scenario 2**. Mrs Turner is an 85-year-old lady who presents to her GP with shortness of breath and knee pain. During his routine examination, the GP notices that Mrs Turner appears to be rubbing her right hand. On further questioning she reports some symptoms around her thumb. The drawing below indicates symptomatic areas shaded by Mrs Turner.

**Figure 3:**
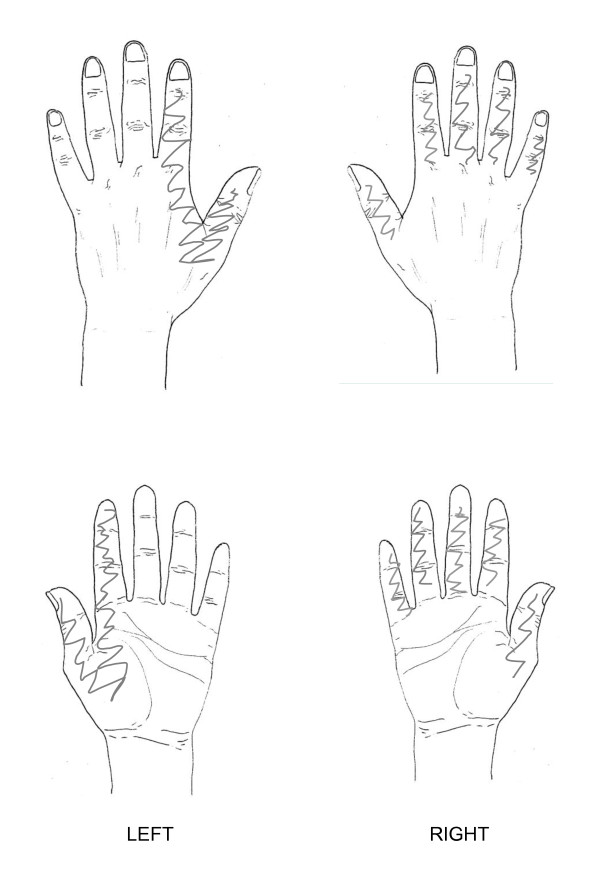
**Case scenario 3**. Mrs Zhu is a 63-year-old lady with a 6-month history of hand symptoms. She expresses concern that her hands have become weaker and she has started to drop things. Mrs Zhu is experiencing difficulty with activities such as dressing, writing, and turning taps on and off. The drawing below indicates symptomatic areas shaded by Mrs Zhu.

Case scenarios were used in this consensus study to provide a context for the participants. Three case scenarios were constructed using anonymised data from people with hand problems [19] to represent community-dwelling older adults with hand problems, and formed the basis of all three rounds of the Delphi survey (figures 1, 2 and 3).

#### Round 1

The aim of Round 1 was to generate a list of questions and assessments suitable for evaluating older adults with self-reported hand problems in primary care. In designing the Delphi study, developmental work using a clinical advisory group had suggested that a framework giving participants some broad headings would be more effective than simply presenting participants with a blank sheet. To control the amount of data produced and to provide a structure for participants, a framework of sections and headings was developed based on the British Association of Hand Therapists (BAHT) Standard on Assessment [20] and using recommendations from the hand therapy literature [21,22]. These headings were then used throughout the Delphi process. The Delphi questionnaire was divided into two sections: 'questions' and 'assessments'. The question section was designed with broad headings: general, pain, stiffness, swelling, sensation, strength, function, and other, under which the Delphi participants were asked to write appropriate questions. The assessment section was also divided into broad headings (observation, palpation, specific tests, examination, and function). Under these headings, participants were requested to use a combination of closed response tick boxes and free text to record their responses.

In order not to make the task too time consuming or repetitive for participants, case scenario one (figure 1), which represented the most severe case, formed the main focus for the participants with the other two scenarios (figures 2 and 3) being supplementary. For the two supplementary case scenarios, participants were asked to indicate which questions and assessments they would include that had not already been addressed in the main case scenario.

#### Round 2

The aim of Round 2 was for participants to rate the importance of responses generated from Round 1 for evaluating the hand. All questions and assessments generated in Round 1 were rated using a nine-point numerical rating scale. A nine-point numerical rating scale was used to allow the participants to make finer judgements then could be made using a simple three-point scale, and to allow a neutral position to be adopted if required. Evidence suggests that five-or seven-point scales produce the most reliable results [23], but as there is a tendency for respondents to avoid the extremities of a scale [24], a nine point scale was chosen. This scale had the additional advantage, that for analysis, it could be divided into three equal categories, overcoming the problems associated with presenting a three point scale to participants, namely a loss of information through limited response options and a related reduction in reliability [23,16,25]. Three verbal descriptors (not important, uncertain, and most important) were used at equal points on the scale to aid interpretation. Evidence suggests that there is little difference between responses to scales with all points labelled and responses to scales with only the ends labelled [26,27]. There is a tendency for scales with verbal anchors at the ends to pull responses towards the ends of the scale [28]. For this reason, a descriptor was also used in the middle of the scale. Importance was further defined for participants as being "of great significance, consequence or value in gaining a clear picture of the nature and severity of hand symptoms, their impact on the person and indications for healthcare".

#### Round 3

The aim of Round 3 was to reduce the number of items retained from Round 2. General cues detailing the length of time available for assessment were added to the case scenarios at this stage. For the main case scenario, participants were asked to choose no more than 40 questions and 20 assessments. For the two supplementary case scenarios, participants were asked to indicate which questions and assessments they would include that had not already been incorporated in the main case scenario.

### Analysis

Content analysis was used to analyse the data generated from Round 1 with each question and assessment being coded by the researcher (HM) and checked by an independent observer (KD). The frequency with which words or terms were used was systematically counted and summarised into categories by identifying common themes in participants' responses. These were summarised under the framework of headings developed for the Delphi study. Prior to analysis it was decided that all questions and assessments generated by participants would be included in the second round.

Data from responses to Round 2 were converted into three categories: ratings 1-3 became category 1 (not important), ratings 4-6 became category 2 (uncertain), and ratings 7-9 became category 3 (most important). Frequencies of the categories for each of the questions and assessments were calculated. Participants' responses were assessed for the degree of consensus based on a decision rule that if two thirds of the participants agreed that an item was "most important" it was retained for Round 3. In the absence of consensus as to the best way of defining agreement, the use of simple decision rules is supported in the literature [16], the definition of which is dictated by the importance of the outcome of the study.

For Round 3, frequencies of positive responses for each of the questions and assessments were calculated. Participants' responses were assessed for the degree of consensus based on a decision rule that if two thirds of the participants agreed that an item should be included it was retained.

## Results

From the twenty-six HCPs who were approached, twenty-two agreed to participate. Response to each stage of the Delphi study is illustrated in table 1.

**Table 1:** Response to each stage of the Delphi study

	Professional group			
	
Stage of study:	OT	Physiotherapist	Rheumatologist	GP
Agreed to participate	13	4	3	2
Completed round 1	7	4	3	2
Completed round 2	5	3	3	2
Completed round 3	4	3	2	2

Figures are numbers of participants

Sixteen participants (73%) responded to Round 1 (table 1) generating a total of 156 questions and 143 assessments (table 2).

**Table 2:** Summary of results from each Delphi round

	**Delphi round 1**	Delphi round 2*	**Delphi round 3^**
	
***Questions***			
Case scenario 1	130	75	24
Case scenario 2	13	9	1
Case scenario 3	13	10	0
**Total**	**156**	**94**	**25**
***Assessments***			
Case scenario 1	123	47	17
Case scenario 2	11	5	1
Case scenario 3	9	3	1
**Total**	**143**	**55**	**19**

*consensus defined as 9 out of 13 participants agreeing that an item was in category 3 (most important) and was subsequently included in Round 3

**^**consensus defined as 7 out of 11 participants agreeing that an item should be included and was subsequently retained at the end of the Delphi study

Thirteen (81%) of the sixteen participants who were mailed responded to Round 2 (table 1). Using the decision rule, 9 or more of the participants rated 94 questions and 55 assessments as being "most important" (table 2).

Responses from those taking part in Round 2 were subject to a small amount of missing data: of the 299 items, 12 were not rated by all thirteen participants. However, had complete data been achieved for these items, the outcome of Round 2 would not have been affected.

Eleven (85%) of the thirteen participants who were mailed responded to Round 3 (table 1). Using the decision rule, 7 or more of the participants agreed that 25 questions and 19 assessments should be included in the evaluation (table 2). These final questions and assessments are presented in table 3.

**Table 3:** Summary of the items retained from the Delphi process.

General questions:
Do you have problems with one or both hands?
Do you have any previous history of the same type of problem?
When did your symptoms start?
How did your symptoms start-were there any identifiable incidents?
Have you ever had surgery or injuries to your hands? If so when and what?
Are your symptoms getting worse, better or about the same since they started?
How is your general health?
Do you have any illnesses e.g. diabetes, heart condition or arthritis?
Do you have any problems anywhere else with your joints or muscles?
Are you on any medication or having any other medical treatment at the moment?
Are you right or left handed?
What have you done so far to get relief?
Have you had to take time off work or stop work because of your symptoms?
**Specific symptom questions:**
Where do you have the pain?
When does the pain occur-at night, with usage, at rest or does it hurt all the time?
What makes it better/worse?
Does pain limit your activities?
Do you experience any thumb pain during activity, e.g. writing, carrying a plate, or turning a key in a lock?
Do you experience stiffness?
Have you noticed any swelling in your hand or puffiness in your fingers?
Do you have any altered sensation (e.g. pins and needles, tingling or numbness) in any part of your hand?
Do you think your strength has decreased?

**Function questions:**
What are you not able to do now that you were able to do before the onset of this problem?
What is involved with your job?

**Other questions:**
Have you had any neck, shoulder or elbow problems-now or in the past?

**Examinations:**
Observation of upper limb/hand posture/use of hand
Observation of swelling
Observation of muscle wasting
Observation of skin condition: colour/pallour/discolouration/cyanosis/redness/Raynaud's
Observation of overall pattern of deformity at rest
Observation of deformity on use-what doesn't work properly
Observation of wrist deformity-subluxed carpus, radial or ulnar deviation, supinated or pronated carpus
Observation of MCP joint deformity-subluxed, radial/ulnar drift, hyperextension
Observation of PIP joint deformity-flexion contracture, hyperextension or lateral deformity, swan neck, Boutonierre
Palpation of swelling
Palpation of pain/tenderness
Palpation of CMC joint/thumb base for pain
Assessment of neck

**Specific tests:**
Phalen's test (carpal tunnel syndrome)

**Evaluation of range of movement:**
Ability to make a full fist
Ability to flatten hand onto a flat surface

**Evaluation of muscle power:**
Power grip strength

**Evaluation of sensation:**
Light touch/threshold testing (e.g. monofilaments/general map)

**Assessment of function:**
Broad hand function-activities of daily living

## Discussion

In this postal Delphi study, a sample of UK HCPs identified a pool of 156 questions and 143 assessments relevant to the evaluation of hand problems in primary care. By the third round, participants agreed on the importance of 25 questions and 19 assessments.

There are many sources of expert advice and guidance on the clinical assessment of musculoskeletal conditions in general and specified single musculoskeletal diagnoses [29-31], but fewer that focus on undifferentiated hand problems as they might present to primary care. Nevertheless, some relatively direct comparisons are possible with the Gait Arms Legs and Spine (GALS) schedule [11], the Regional Examination of the Musculoskeletal System (REMS) for medical students [12], and the Southampton examination schedule for diagnosis of specific upper limb musculoskeletal disorders [13,32]. These existing schedules were not considered during the development of the framework for the Delphi rounds as the hand assessment component was either concerned mainly with observation and palpation, or the focus was on diagnostic classification and screening, and as such, we did not wish to replicate work which had already been done. Although previous studies suggest that in older people osteoarthritis is likely to be the most common condition, we wanted Delphi participants to think more broadly-about undifferentiated hand problems in primary care-rather than biasing participants towards a particular diagnostic group, which using these schedules within the development of the framework may have done. There were however similarities between the findings from this Delphi study and the hand assessment component of the GALS [11], the REMS [12], and the Southampton schedule [13,32]. In particular, the emphasis on observation of gross movement (for example, ability to make a full fist) in contrast to instrumented measurement of movement at individual joints was evident in both the GALS and this Delphi study. However, the assessment of joint nodes and bony enlargements, whilst included in the GALS, REMS and the Southampton schedule, was omitted by the Delphi participants. Presence of these features is indicative of hand OA; a condition that is generally considered by HCPs to be an inevitable part of ageing and as such, more limited in treatment options and less serious than inflammatory arthropathies [33,34]. The final examinations agreed by the Delphi participants (observation and palpation of swelling, palpation of pain/tenderness and observation of deformity) appear to be targeted at identifying more serious but treatable diseases such as inflammatory arthritis.

Limited comparisons can be made between the results from this Delphi study and national guidelines for the assessment of OA published after this study was undertaken [35]. Although these are not specific to the assessment of the hand they do suggest that the assessment of OA ought to be 'holistic' including consideration of activities of daily living, hobbies and occupation. Indeed, items relating to all of these categories were generated in the Delphi study but not retained in the final selection by the Delphi participants. Generally, Delphi participants chose more items related to symptoms (pain, stiffness, swelling, altered sensation and weakness) than function. The Delphi participants agreed that only one question and three assessments relating to hand function should be retained at the end of the study. Standardised hand function tests were excluded in the second round in favour of assessment of broad hand function. This may be due to a genuine lack of agreement about the relative importance of the many different functional questions and tests available. It may also reflect what previous authors have noted: namely, that for busy clinicians, time constraints and lack of knowledge about hand function tests may preclude the use of a standardised test of hand function [36,37].

In the absence of empirical evidence, this study has provided an indication of current opinion on the most important questions and assessments for evaluating the hand in primary care. The Delphi technique used in this study achieved its aim of generating a comprehensive list of questions and assessments and filtering them into a core of essential items. Delphi participants were chosen purposively to represent the main professional groups involved in musculoskeletal hand assessment, although others of the same professional background may not necessarily share the views of the individuals who participated in this study. A good response rate was achieved for each round, exceeding the 70% recommended [38]. Although attrition occurred at each stage of the Delphi study, the final number of participants satisfied the need to have 10 or more participants to achieve good reliability [16]. Response rates for this study are comparable to others [18,39-42].

Although the Delphi is a useful tool for identifying consensus, it is not without its limitations. While the intention of this study was to identify the important components of the assessment of hand problems in older adults within primary care, the HCPs approached and recruited to the study were not all specifically involved in the delivery of front line care. These participants were purposively selected as the majority of health practitioners with experience and specialist skills in assessing the hand tend to be employed in a secondary care setting. Inclusion of other HCPs with specialist hand assessment skills working in secondary care, such as hand surgeons, may have provided a different perspective from that of the other participants on the Delphi panel. Evidence suggests, that whilst the prevalence of hand problems within primary care is high, consultation with HCPs is low [3], suggesting that front line HCPs may be likely to have less experience of assessing hands than their secondary care counterparts. The high prevalence of inflammatory athropathies in secondary care settings is reflected in the outcome of this Delphi study. For example, items relating to examination of deformity were primarily concerned with the deformities that would occur with inflammatory arthritis.

Despite attempts to minimise attrition, participants dropped out at each stage of the study. Attrition was greatest amongst the OTs, particularly between the point of agreeing to participate in the study and returning the first round of the Delphi questionnaire. However, after this, the rate of attrition amongst the OTs was generally comparable to the other professional groups, with the exception of the GPs, for whom there was no attrition. The early attrition could therefore be considered as a reflection of recruitment rates.

The sample size could be considered a limitation of this study, although for Delphi studies where the purpose of the study is focussed and the participants are from similar backgrounds, it is recommended that 15-20 participants should be recruited [17]. Studies with 20 or fewer participants have the advantage of being more successful than larger studies in reducing attrition rates [28].

Future work will establish the reliability, feasibility and value of using these questions and assessments identified for older adults with hand problems in a primary care setting. Specifically, the relative contribution of these questions and assessments in evaluating the nature and severity of hand problems, assisting diagnosis, indicating appropriate management, and predicting future outcome requires further investigation.

## Conclusions

We describe a consensus study, using a Delphi technique with HCPs, to identify core questions and assessments for use in primary care with older adults reporting hand pain or hand problems. The Delphi study generated a pool of items and identified those which were perceived to be the most important by a majority of participants for assessing this group. However, the actual importance, usefulness and relevance of these items cannot be judged from the Delphi study alone. The long-term aim of this work is to determine the relationship between clinical questions and assessments and the clinical course of hand pain and hand problems in community-dwelling older adults. The next step in achieving this will be to establish the reliability of the clinical questions and assessments retained at the end of this Delphi study.

## Competing interests

The authors declare that they have no competing interests.

## Authors' contributions

All authors contributed to the conception and design, execution, analysis and interpretation of data, were involved in drafting and critically revising the article, and read and approved the final version.

## Pre-publication history

The pre-publication history for this paper can be accessed here:

http://www.biomedcentral.com/1471-2474/11/178/prepub
